# Older age is associated with more positive reframing of memories from the spring phase of the covid-19 pandemic

**DOI:** 10.1093/geroni/igaa057.3490

**Published:** 2020-12-16

**Authors:** Sandry Garcia, Jaclyn Ford, Eric Fields, Tony Cunningham, Elizabeth Kensinger

**Affiliations:** 1 Boston College, Brookline, Massachusetts, United States; 2 Boston College, Chestnut Hill, Massachusetts, United States; 3 Boston College, Chestnut Hill, United States

## Abstract

Older adults often focus on positive aspects of past events, showing resiliency that runs counter to negative stereotypes of aging. We asked whether older adults would retain this positive focus even during the COVID-19 pandemic that puts older adults at particular health risk. We examined how age would relate to the experience of affect during the spring phase of the pandemic and also how it would affect their memories of that spring phase. We predicted that when reflecting on the spring phase of the pandemic, older age would be associated with an increased tendency to focus on “silver linings” and a decreased focus on negative aspects. Furthermore, we explored whether focusing on those “silver linings” would be driven by (i) more positive experienced affect during the spring-phase, or (ii) by a memory-specific effect that would persist after controlling for experienced affect. As predicted, older age was associated with increased focus on “silver linings” such as feelings of hope that the measures would reduce disease spread (p <.001) or memories of the community coming together (p <.001). These effects remained even when controlling for older adults’ more-positive affect during the spring phase of the pandemic (community coming together, p=.03, and feeling hope, p=.008). These results suggest that older adults remain resilient during the ongoing pandemic. Compared to younger adults, older adults experience greater affective well-being in the moment and also benefit from a memory-specific mechanism allowing them to view the ongoing negative event through a rosier lens.

